# Targeting critical kinases and anti-apoptotic molecules overcomes steroid resistance in MLL-rearranged leukaemia

**DOI:** 10.1016/j.ebiom.2021.103235

**Published:** 2021-02-10

**Authors:** Anne P. de Groot, Yoriko Saito, Eiryo Kawakami, Mari Hashimoto, Yuki Aoki, Rintaro Ono, Ikuko Ogahara, Saera Fujiki, Akiko Kaneko, Kaori Sato, Hiroshi Kajita, Takashi Watanabe, Masatoshi Takagi, Daisuke Tomizawa, Katsuyoshi Koh, Mariko Eguchi, Eiichi Ishii, Osamu Ohara, Leonard D. Shultz, Shuki Mizutani, Fumihiko Ishikawa

**Affiliations:** a Laboratory for Human Disease Models, RIKEN Center for Integrative Medical Sciences, 1-7-22 Suehiro-cho, Tsurumi-ku, Yokohama, Kanagawa 230-0045, Japan; b Healthcare and Medical Data Driven AI based Predictive Reasoning Development Unit, RIKEN Medical Sciences Innovation Hub Program, Yokohama, Japan; c Department of Pediatrics, National Cancer Center Hospital, Tokyo, Japan; d Laboratory for Integrative Genomics, RIKEN Center for Integrative Medical Sciences, Yokohama, Japan; e Department of Pediatrics and Developmental Biology, Tokyo Medical and Dental University, Tokyo, Japan; f Division of Leukaemia and Lymphoma, Children's Cancer Center, National Center for Child Health and Development, Tokyo, Japan; g Department of Hematology/Oncology, Saitama Children's Medical Center, Saitama, Japan; h Department of Pediatrics, Ehime University Graduate School of Medicine, Ehime, Japan; i Kazusa DNA Research Institute, Kisarazu, Chiba, Japan; j The Jackson Laboratory, Bar Harbor, USA

**Keywords:** Humanized mouse, MLL, Leukaemia, Apoptosis, Xenograft

## Abstract

**Background:**

Acute lymphoblastic leukaemia with mixed lineage leukaemia gene rearrangement (MLL-ALL) frequently affects infants and is associated with a poor prognosis. Primary refractory and relapsed disease due to resistance to glucocorticoids (GCs) remains a substantial hurdle to improving clinical outcomes. In this study, we aimed to overcome GC resistance of MLL-ALL.

**Methods:**

Using leukaemia patient specimens, we performed bioinformatic analyses to identify target genes/pathways. To test inhibition of target pathways in vivo, we created pre-clinical therapeutic mouse patient-derived xenograft (PDX)-models by transplanting human MLL-ALL leukaemia initiating cells (LIC) into immune-deficient NSG mice. Finally, we conducted B-cell lymphoma-2 (BCL-2) homology domain 3 (BH3) profiling to identify BH3 peptides responsible for treatment resistance in MLL-leukaemia.

**Findings:**

Src family kinases (SFKs) and Fms-like tyrosine kinase 3 (FLT3) signaling pathway were over-represented in MLL-ALL cells. PDX-models of infant MLL- ALL recapitulated GC-resistance in vivo but RK-20449, an inhibitor of SFKs and FLT3 eliminated human MLL-ALL cells in vivo, overcoming GC-resistance. Further, we identified BCL-2 dependence as a mechanism of treatment resistance in MLL-ALL through BH3 profiling. Furthermore, MLL-ALL cells resistant to RK-20449 treatment were dependent on the anti-apoptotic BCL-2 protein for their survival. Combined inhibition of SFKs/FLT3 by RK-20449 and of BCL-2 by ABT-199 led to substantial elimination of MLL-ALL cells in vitro and in vivo. Triple treatment combining GCs, RK-20449 and ABT-199 resulted in complete elimination of MLL-ALL cells in vivo.

**Interpretation:**

SFKs/FLT3 signaling pathways are promising targets for treatment of treatment-resistant MLL-ALL. Combined inhibition of these kinase pathways and anti-apoptotic BCL-2 successfully eliminated highly resistant MLL-ALL and demonstrated a new treatment strategy for treatment-resistant poor-outcome MLL-ALL.

**Funding:**

This study was supported by RIKEN (RIKEN President's Discretionary Grant) for FI, Japan Agency for Medical Research and Development (the Basic Science and Platform Technology Program for Innovative Biological Medicine for FI and by NIH CA034196 for LDS. The funders had no role in the study design, data collection, data analysis, interpretation nor writing of the report.

**Research in context**

**Evidence before this study**

Acute lymphoblastic leukaemia (ALL) with rearrangement of the mixed-lineage leukaemia (MLL) gene frequently affects infants and is associated with a poor prognosis due to their poor response to the standard treatment in ALL with glucocorticoids. The Fms-like tyrosine kinase 3 (FLT3) is overexpressed in MLL-ALL and Src-family kinases (SFKs) activation account for GC-resistance in MLL-ALL. Therefore, these kinases are considered as potential drug-targets in MLL-ALL.

**Added value of this study**

We created a pre-clinical therapeutic mouse model by transplanting human MLL-ALL leukaemia initiating cells into immune-deficient mice. This model recapitulated GC-resistance. GC treatment combined with dual inhibition of FLT3 and SFKs with our previously developed compound RK-20449 led to reduction of GC-resistant human leukaemia cells in the MLL-ALL engrafted mice. Furthermore, we found that MLL-ALL cells resistant to RK-20449 treatment were dependent on the anti-apoptotic B-cell lymphoma (BCL-2) protein for their survival and they could be eliminated from the MLL-ALL engrafted mice by additional inhibition of BCL-2 with ABT-199.

**Implications of all the available evidence**

This study demonstrates that inhibition of FLT3 and SFKs overcomes GC-resistance in MLL-ALL engrafted mice. Further, this study demonstrates that triple-treatment with GCs, RK-20449 and ABT-199 completely eliminates leukaemia cells from the MLL-ALL engrafted mice. These results suggest that combined inhibition of kinases and anti-apoptotic proteins targets MLL-ALL cells and therefore this study identified a new potential drug-treatment strategy for infant MLL-ALL patients.

Alt-text: Unlabelled box

## 1 Introduction

Acute lymphoblastic leukaemia (ALL) is the most common type of leukaemia in children [1]. ALL is characterized by an uncontrolled proliferation of malignant lymphoid cells in the bone marrow (BM) accompanied by suppression of other haematopoietic lineages. Fortunately, the overall complete remission rate for childhood ALL has increased significantly in the last decades and has recently been reported as >95% [1]. Among various childhood ALL, mixed-lineage leukaemia (MLL) gene-rearranged ALL (MLL-ALL) is associated with one of the worst prognoses in which 5-year survival rates remain approximately 40% [[2], [3], [4], [5]]. Though recent effort in risk stratification and intensified chemotherapy and stem cell transplantation improved clinical outcome to a certain extent [6,7] treatment resistance of MLL-ALL and MLL-AML has been one of the critical issues to be addressed [8]. The MLL (also known as KMT2A) gene encodes a histone modifying enzyme that catalyzes a specific lysine 4 (H3K4), which is essential for the lineage commitment of haematopoietic stem and progenitor cells (HSPCs) [9,10]. Chromosomal translocation between the MLL gene and a fusion partner gene, leading to production of abnormal fusion proteins, results in disturbed hematopoiesis of HSPCs. Among over 70 fusion partners reported, translocations involving AF4, AF9 and ENL are the most frequent in MLL-ALL [11,12]. These fusion partners of MLL are responsible for the sustained overexpression of Hox genes and drives MLL-ALL proliferation [13,14] .

Currently, glucocorticoids (GCs), such as prednisone and dexamethasone, in combination with conventional chemotherapeutic agents such as cytarabine, etoposide, or daunorubicin are used to treat infant ALL. Although GC is a key agent for targeting lymphoid malignancies, basic experimental studies showed that MLL-ALL cells become rapidly resistant to GCs *in vitro* [[15], [16], [17], [18], [19], [20], [21], [22]] an *in vivo* [3,18,20,21]. Therefore, it is important to develop new drugs that can recognize the abnormal proteins, expressed by MLL-ALL cells and subsequently eliminate these. Armstrong and colleagues showed that infant MLL-ALL cells express higher levels of Fms-like tyrosine kinase 3 (FLT3) compared to MLL-germline ALL and that high expression of FLT3 in MLL-ALL correlates with poor prognosis [13]. Furthermore, FLT3 is constitutively activated in MLL-ALL cells [23]. As another potential mechanism for GC-resistance, phosphorylation of Src-family kinases (SFKs) together with high expression of annexin A2 may lead MLL-ALL cells resistance to GCs. Previous reports supported the involvement of SFKs in treatment resistance of MLL-ALL cells to GCs by showing that inhibition of SFKs prevented and reversed GC-resistance in MLL-ALL cells *in vitro* [20,21,24].

In this study, we examined the effect of dual inhibition of FLT3 and SFK pathways in GC-resistant infant MLL-ALL *in vivo* using a small molecule inhibitor RK-20449 [25]. Using MLL-ALL patient-derived xenograft (PDX) models that we have previously reported [26], we found addition of RK-20449 to dexamethasone eliminated GC-resistant primary MLL-ALL cells *in vivo* in a majority of recipients engrafted with infant MLL-ALL. However, in some cases, MLL-ALL cells were significantly reduced but not completely eliminated. Through B-cell lymphoma 2 (Bcl-2) homology domain 3 (BH3) profiling, we found that these resistant cells were dependent on Bcl-2 for survival and combined treatment using dexamethasone, FLT3/SFK inhibition by RK-20449 and Bcl-2 inhibition by ABT-199 completely eradicated human MLL-ALL cells both *in vitro* and *in vivo*.

## 2 Methods

### 2.1 Human samples ethics

The study using patient specimens has been approved at RIKEN Institutional Review Board (approval number:17-17-4 [3]). 15 different MLL-ALL patients BM or peripheral blood (PB) samples were obtained from Saitama Children's Medical Center (*n*=6; Pt.1,4,6,7,13,20, collected on 5th August 2011, 16th June 2011, 14th July 2010, 12th May 2011, 10th October 2013, 3rd June 2014, respectively), Shizuoka Children's Hospital (*n*=1; Pt.2 collected on 13th July 2011), Tokyo Medical and Dental University, Medical Hospital (*n*=5; Pt.3,5,9,10,14 collected on 4th June 2010, 19th February 2010, 4th June 2010, 4th June 2008, 4th June 2010, respectively), University of Tsukuba Hospital (*n*=1; Pt.8 collected on 28th July 2011), Niigata Cancer Center Hospital (*n*=1; Pt.11 collected on 22nd July 2011), and Ehime University (*n*=1; Pt.12 collected on 6th December 2011). Five non-MLL ALL patient samples were collected from Tokyo Medical and Dental University, Medical Hospital (*n*=3; Pt.15,16,17 collected on 25th December 2013, 12th September 2013, 4th September 2013, respectively), Kochi Health Sciences Center *(n*=1; Pt.18 collected on 19th December 2012), and Ehime University (*n*=1; Pt.19 collected on 7th December 2011). All participants gave written informed consent in accordance with the Declaration of Helsinki. Normal CB were obtained from Chubu Cord Blood Bank.

### 2.2 Mice ethics

NOD.Cg-*Prkdc^Scid^Il2rg^tmlWjl^*/Sz (NSG) mice were bred and maintained under defined flora at the animal facility of RIKEN and at The Jackson Laboratory according to guidelines established by the Institutional Animal Committees at each institution. The approval number for our mouse experiments at RIKEN is 2020-019 [3]. We followed the ARRIVE guidelines in every experiment. Transplantation experiments were performed using 1–3 day old NSG mice.

### 2.3 Transplantation

For xenogeneic transplantation, cells were obtained from BM or PB of ALL patients. Mononuclear cells were isolated by density centrifugation (Lymphocyte Separation Medium, MP Biomedicals), then labeled with mouse monoclonal antibodies to human CD34, CD38, CD19, CD33, and CD3 (BD Biosciences), followed by isolation of CD3-CD33-CD34+CD38+CD19+ and CD3-CD33-CD34-CD19+ cells by cell sorting using FACSAria III (BD). 10^2^ to 10^5^ purified cells were injected IV in NSG newborns after 1.5 G total body irradiation. For serial transplantation, mouse CD45 (mCD45)- mouseTer119- human CD45 (hCD45)+ cells isolated from recipient BM or spleen were used as donor cells.

### 2.4 *In vivo* treatment

MLL-ALL-engrafted NSG recipients underwent *in vivo* treatment using dexamethasone 30 mg/kg i.p. once daily (SIGMA), RK-20449 30 mg/kg i.p. twice daily [25], ABT-199 (Active Biochem) 30 mg/kg p.o. once daily and combination drug treatment as indicated. During treatment, we harvested 20 μL PB from each mouse once per week to determine hCD45+ leukemic cell chimerism. Recipient mice were euthanized after 4–7 weeks of treatment or when they became moribund.

### 2.5 Flow cytometry analysis

Recipient PB, BM and spleen of the recipient mice were stained with fluorochrome-conjugated monoclonal antibodies to mTer119 (Clone TER119, RUO), mCD45 (Clone HI30, RUO), hCD45 (Clone 30-F11, RUO), CD3 (Clone UCHT1, RUO), CD19 (Clone SJ25C1, RUO), CD33 (Clone WM53, RUO), CD34 (Clone 8G12, RUO(GMP)), and CD38 (Clone HB7) (BD Biosciences) and analyzed using FACSAria III or FACSCanto II (BD) and Flowjo software v10.4.0.

### 2.6 Microarray

MLL and non-MLL leukaemia samples were obtained through JPLSG (Japan Pediatric Leukaemia/Lymphoma Study Group). Previously published microarray data [26] and new microarray data were used for transcriptome analysis. In short, transcriptome data of CD34- or CD34+CD38+ MLL-leukaemia initiating cells were obtained from 14 different MLL-ALL patients (Pt.1,4,6,7,13,20 collected on 9th August 2011, 14th July 2011, 17th May 2011, 14th July 2011, 23rd March 2020, 23rd March 2020, respectively, from Saitama Children's Medical Center; Pt.3,5,9,10,14 collected on 17th May 2011 from Tokyo Medical and Dental University, Medical Hospital; Pt.2 collected on 7th September 2011 from Shizuoka Children's Hospital; Pt.11 collected on 7th September 2011 from Niigata Cancer Center Hospital; Pt.12 collected on 23rd March 2020 from Ehime University). For non-MLL leukaemias, transcriptome data of ALL-initiating cells were obtained on 23rd March 2020 from one Philadelphia chromosome-positive (Ph+) ALL patient (CD34-CD19+ for Pt.15, Tokyo Medical and Dental University, Medical Hospital), two different ETV6-RUNX1 translocated patients (CD34+CD19+ for Pt.16 and 17 from Tokyo Medical and Dental University, Medical Hospital), one t(5;15) translocated patient (CD34-CD19+ for Pt.18 from Kochi Health Sciences Center), and one patient without cytogenetic profile (CD34+CD19+ for Pt.19 from Ehime University). Normal BM cells were purchased from Cambrex (Walkerville). RNA was extracted using TRIzol (Invitrogen) and cDNA was amplified by using ovation RNA Amplification System V2 Kit (NuGEN). The cDNA was fragmented and labeled for Human Genome U133 plus 2.0 GeneChip (Affymetrix). Microarray data was analyzed by R Studio 1.1.463 [27] using the Bioconductor package (http://www.bioconductor.org/). Analyzed data was normalized for probe signal intensity using the GeneChip Robust Multiarray Averaging (GC-RMA) package [28] and the Limma package [29] was used for identifying differently expressed genes (DEGs) between ALL patient samples and normal BM haematopoietic stem cells (HSCs). The STRING data base [30] was used to find interactions among the DEGs (using high confidence score > 0.700, clusters were tweaked) and visualized with Cytoscape 3.7.2 [31]. Pathway enrichment analysis was performed with the ClusterProfiler package [32] using the “Kyoto Encyclopedia of Genes and Genomes (KEGG)” database [33] and visualized with Cytoscape 3.7.2. Heat maps showing transcript levels in MLL and non-MLL leukaemias were created using the heatmap package [34].

### 2.7 Flow cytometric analysis for phospho-kinase protein

2 × 10^6^ BM cells obtained from MLL-ALL engrafted recipients (MLL-AF4; Pt.2, 4, MLL-ELN; Pt.8, MLL-AF9; Pt.10, 11, 12, 13), non-MLL ALL engrafted recipients (Ph+ ALL; Pt. 15, TEL-AML; Pt.16, t(5;15); Pt.18, unknown karyotype; Pt.19) or freshly isolated normal CD34+ cord blood cells were fixed using Lyse/Fix buffer 5x (BD, 558049) at 37 °C for 10 min then permeabilized using Phosflow Perm Buffer III (BD, 558050) at −30 °C for 30 min. Non-specific background was blocked with BSA stain buffer (BD, 554657) and Fc receptors were blocked by incubating cells in 1% mouse Fc Block (BD, 553142) at 4 °C for 5 min. Cells were then stained for surface markers hCD45, mCD45 and intracellular proteins pNF-κB (pS529), pAkt (pS473), pS6 (pS235/pS236) and p4EBP1 (pT36/pT45) (BD) at 4 °C for 1 h and analyzed using FACSCanto II (BD).

### 2.8 Immunohistochemistry

Tissue sections (3 μm) were cut from 4% paraformaldehyde (PFA)-fixed paraffin-embedded recipient organs. Sections were deparaffinized using xylene and ethanol and antigen retrieval was performed (Retrievagen A (pH 6.0), BD Pharmingen^TM^). Non-specific background was reduced by incubating the slides in methanol + H_2_O_2_ (Wako). After blocking with horse serum, slides were incubated with mouse anti-human CD45 antibody (DAKO, M0701) (1:150) then HRP-conjugated horse anti-rabbit/mouse IgG antibody (ImmPRESS^TM^, Cat. MP-7500). Slides were then stained with 3,3’-diaminobenzine (DAB) and hematoxylin, dehydrated, mounted using Vectamount (Vector Laboratories) and analyzed with Axiovert 200 microscope (Zeiss). Photos were taken using AxioCam MRc 5 (Zeiss) using the AxioVision rel. 4.6 software.

### 2.9 BH3 profiling

BH3 profiling was performed using the protocol described by the Letai lab [35]. In short, 5 × 10^6^ BM cells obtained from recipients engrafted with MLL-ALL were stained with Zombie NIR (BioLegend) for 20 min at RT to exclude dead cells, labeled with surface markers mCD45, hCD45, human CD33, and human CD19 for 30 min at 4 °C. As a control, normal CB cells were used. After surface labeling, cells were resuspended in DTEB buffer (135 mM trehalose, 20 µM EDTA, 20 µM EGTA, 5 mM succinic acid, 0.1% BSA, 10 mM HEPES and 50 mM KCl) to protect cells from spontaneous cytochrome C release. Cells were then permeabilized (0.001% digitonin) and exposed to BH3-only peptides (0.781 µM BIM, 80 µM NOXA, 80 µM HRK or 80 µM BAD) in DTEB buffer for 1 h. DMSO was used as a negative control and alamethicin (25 µM) as a positive control. Cells were then fixed (4% PFA), neutralized (Buffer-N2), permeabilized (Perm/wash buffer I, BD) and stained for intracellular cytochrome c, using Alexa Fluor® 647 Mouse anti-Cytochrome c (BD).

### 2.10 *In vitro* treatment

For *in vitro* culture, 1-2 × 10^5^ hCD45+ cells isolated from MLL-ALL-engrafted recipient spleen and human CD34+CD38- HSCs by cell sorting were seeded per well in 96-wells plate in Stemline II Haematopoietic Stem Cell Expansion Medium (SIGMA) supplemented with stem cell factor (50 ng/ml), FLT3 ligand (50 ng/ml), thrombopoietin (50 ng/ml), IL-2 (5 ng/ml), IL-3 (20 ng/ml), IL-7 (20 ng/ml) and IL-15 (10 ng/ml). The cells were exposed to 30 nM dexamethasone (SIGMA) or RK-20449 [25] or increasing concentrations of ABT-199 (1 nM, 3 nM, 10 nM and 30 nM) (active Biochem) as indicated for 3 days at 37 °C in humidified atmosphere containing 5% CO_2_. Cells were harvested and stained with BV421-labeled anti-hCD45 and 7AAD, collected in BD TruCount^TM^ Tubes and analyzed using FACSCanto II (BD).

### 2.11 Statistical analysis

For *in vivo* treatment experiments, difference in the percentages of hCD45+ cells in Pre- and Post-treatment PB and in the BM and spleen between the four treatment groups was analyzed using a two-tailed *T*-test whereby *p*<0.05 was considered as a significant difference. Percentages are given as means ± SEM. All analyses were performed by IBM SPSS Statistics v23 software (IBM Corporation ®). Synergistic effects of combination treatment on survival were analyzed using an algorithm based on Bliss definition [36].

### 2.12 Role of the funding source

The funder of the study had no role in study design, data collection, data analysis, data interpretation, or writing of the report. The corresponding authors had full access to all the data in the study and had final responsibility for the decision to submit for publication.

## 3 Results

### 3.1 MLL-ALL patient-derived PDX recapitulates glucocorticoid resistance *in vivo*

**Fig. 1.**
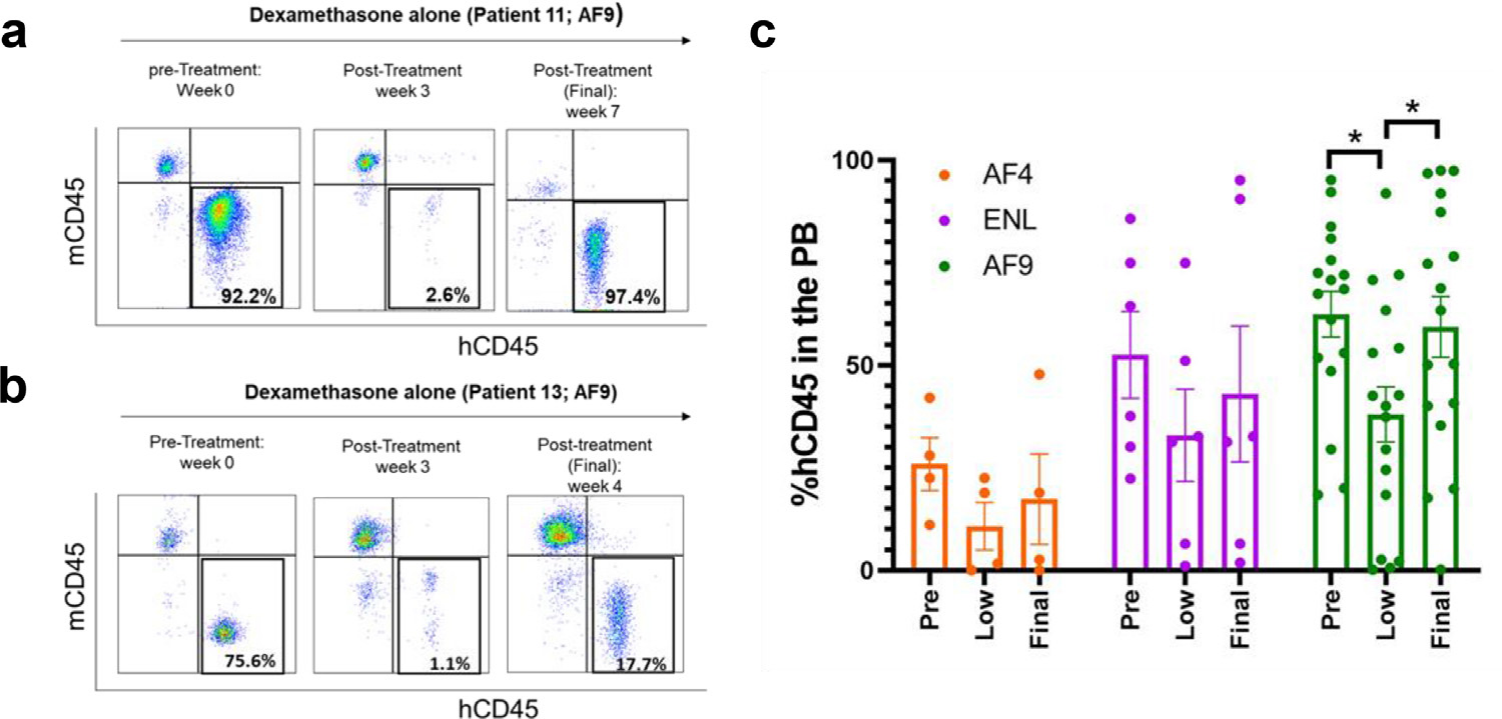
**Glucocorticoid resistance of human MLL-rearranged ALL cells.***In vivo* effect of dexamethasone on hCD45+ MLL-ALL cells in the PB of recipient mice treated for 4 to 7 weeks with 30mg/kg dexamethasone once per day. Recipients engrafted with MLL-ALL cells were prepared from three different MLL-AF4 patients, three MLL-ENL patients, and five MLL-AF9 patients (see Table S1). AF4 recipients: *n*=4 (Pt.1 *n*=1, Pt. 2 *n*=2, Pt. 4 *n*=1); ENL recipients *n*=6 (Pt. 6 *n*=3, Pt. 7 *n*=1, Pt. 8 *n*=2); AF9 recipients n=17 (Pt. 10 *n*=3, Pt. 11 *n*=4, Pt. 12 *n*=4, Pt. 13 *n*=5, Pt. 14 *n*=1). (a and b) Flow cytometry plots showing percentages of human and mouse CD45+ cells in PB from AF9 Patients (a) 11 and (b) 13 during dexamethasone treatment period. (c) PB chimerism of human MLL-ALL cells at the start of treatment, at lowest hCD45+ chimerism reached during treatment and at the end of treatment in MLL-ALL recipients. Results are expressed as ± 1 standard error of the mean with *P<0.05 by two-tailed *T*-test.

We created PDX models for glucocorticoid-resistant primary human MLL-ALL by intravenously injecting CD34-CD38+ leukaemia-initiating cells (LICs) from five MLL-AF9 patients, CD34+ or CD34- LICs from three MLL-ENL patients, and CD34+38+ or CD34-38+ LICs from three MLL-AF4 patients into immunocompromised NSG newborns. Patient characteristics are summarized in Table S1. These patient samples were included in our previous publication [26]. We started *in vivo* treatment of MLL-ALL xenografts with dexamethasone (30 mg/kg intraperitoneally once daily) when hCD45+ ALL cell chimerism reached 20% or higher in the recipient PB, then followed PB human ALL chimerism by flow cytometry every week thereafter. Although we found variable resistance of leukemic cells to dexamethasone, leukemic cells proliferated in a majority (16 out of 27) of MLL-ALL samples examined. Overall, we observed that dexamethasone treatment led to only a transient decrease in hCD45+ MLL-ALL cell chimerism, recapitulating human treatment resistance (Fig. 1). To overcome glucocorticoid resistance in MLL-ALL, we aimed to find additional therapeutic targets.

### 3.2 Identification of critical pathways in glucocorticoid-resistant MLL-ALL cells

**Fig. 2.**
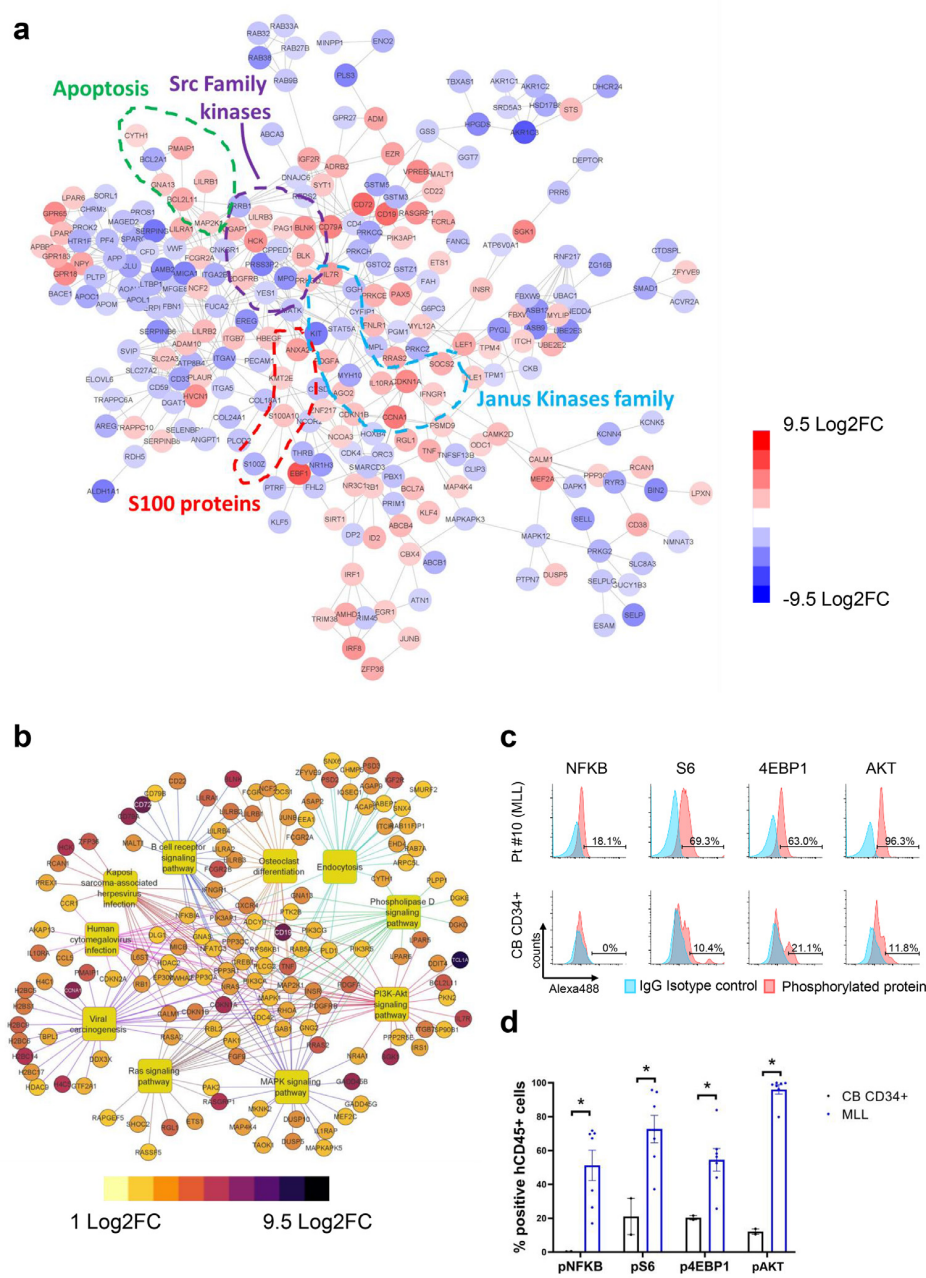
**Different gene expression profile in MLL-ALL patient LICs and normal human HSCs.** (a) Protein-protein interactions among the DEGs (log_2_FC>1 or log_2_FC<-1, and *P* value <0.01) between three independent normal human HSC samples and LICs from MLL-AF4 (Pt. 1–5), MLL-ENL (Pt. 6, 7 and 9), MLL-AF9 (Pt. 10, 11 and 14) patients found by the STRING database. Blue are downregulated and red are upregulated genes. (b) Cnetplot showing both the most enriched pathways and their associated upregulated genes in LICs compared to normal HSCs found by KEGG pathway enrichment analysis. (c and d) Phosphorylation levels of NF-κB, S6, 4EBP1 & AKT in two freshly isolated normal CD34+ cord blood samples or in hCD45+ MLL-ALL cells from NSG-recipients of two AF4 patients (Pt. 2 and 4), one ENL patient (Pt. 8) and four AF9 patients (Pt. 10, 11, 12 and 13) are shown. (c) Representative flow histogram plots showing percentages of phosphorylated proteins in hCD45+ recipient BM cells, using IgG isotype to set a threshold. (d) Phosphorylation levels in hCD45+ MLL-ALL cells obtained from NSG-recipients or normal CD34+ cord blood cells (*n*=7 and 2, respectively). Results are expressed as ± 1 standard error of the mean with *P<0.05 by two-tailed *T*-test.

To identify additional drug targets, we first compared transcriptional profiles of normal BM CD34+CD38- HSCs and MLL-ALL patient-derived LICs. Using our previously published [26] Affymetrix Human Genome U133 array data, we performed a differential expression analysis and identified 915 significant differentially expressed genes (DEGs), of which 301 were upregulated and 614 downregulated in MLL-ALL LICs compared to normal HSPCs. We searched for interactions among proteins encoded by the DEGs using the STRING database [30], and found clusters of proteins associated with apoptosis, Src family kinases (SFKs), Janus Kinases (JAK) family, and S100 proteins (Fig. 2a). Among the proteins associated with SFK signaling pathway, we found HCK, BLK, ANXA2, and S100A10 transcripts to be overexpressed in MLL-ALL cells. Interestingly, we found IL7R, an activator of the JAK-STAT signaling pathway, to be overexpressed in MLL-ALL cells (Fig. 2a). Non-MLL leukaemias, such as Ph+ ALL, ETV6-RUNX1 translocated ALL and t(5;15) translocated ALL, showed comparable transcript profiles as MLL-ALL patients. Similar to MLL-ALL patients, HCK, ANXA2 and IL7R transcripts were overexpressed in non-MLL compared with HSPCs (Fig. S1a). In addition, we performed Kyoto Encyclopedia of Genes and Genomes (KEGG) pathway analysis on the DEGs and determined the top 10 most enriched pathways based on the number of genes involved. Fig. 2b shows both the enriched pathways and the associated overexpressed genes in MLL-ALL cells. Interestingly, several kinases signaling pathways, like PI3K-Akt, MAPK, Ras are enriched in MLL-ALL (Fig. 2b). Though we did not find over-representation of NFKB1, RPS6, EIF4EBP1, and AKT1 genes in MLL ALL at RNA levels as compared with normal CD34+ HSPCs, we found high levels of pNF-κB, pS6, p4EBP1 and pAKT in human MLL-ALL cells at the protein level suggesting functional activation of SFK and/or other kinase signaling pathways (Fig. 2c, d, S1b and S1c). Based on these results, we hypothesized that activation of these kinases contributes to glucocorticoid-resistance in human MLL-ALL cells. To test this hypothesis, we went on to determine whether human MLL-ALL is sensitive to combined dexamethasone and RK-20449 (a small molecule inhibitor of SFKs and FLT3) treatment *in vivo*.

### 3.3 Human MLL-ALL cells are eliminated by RK-20449 in combination with dexamethasone *in vivo*

**Fig. 3.**
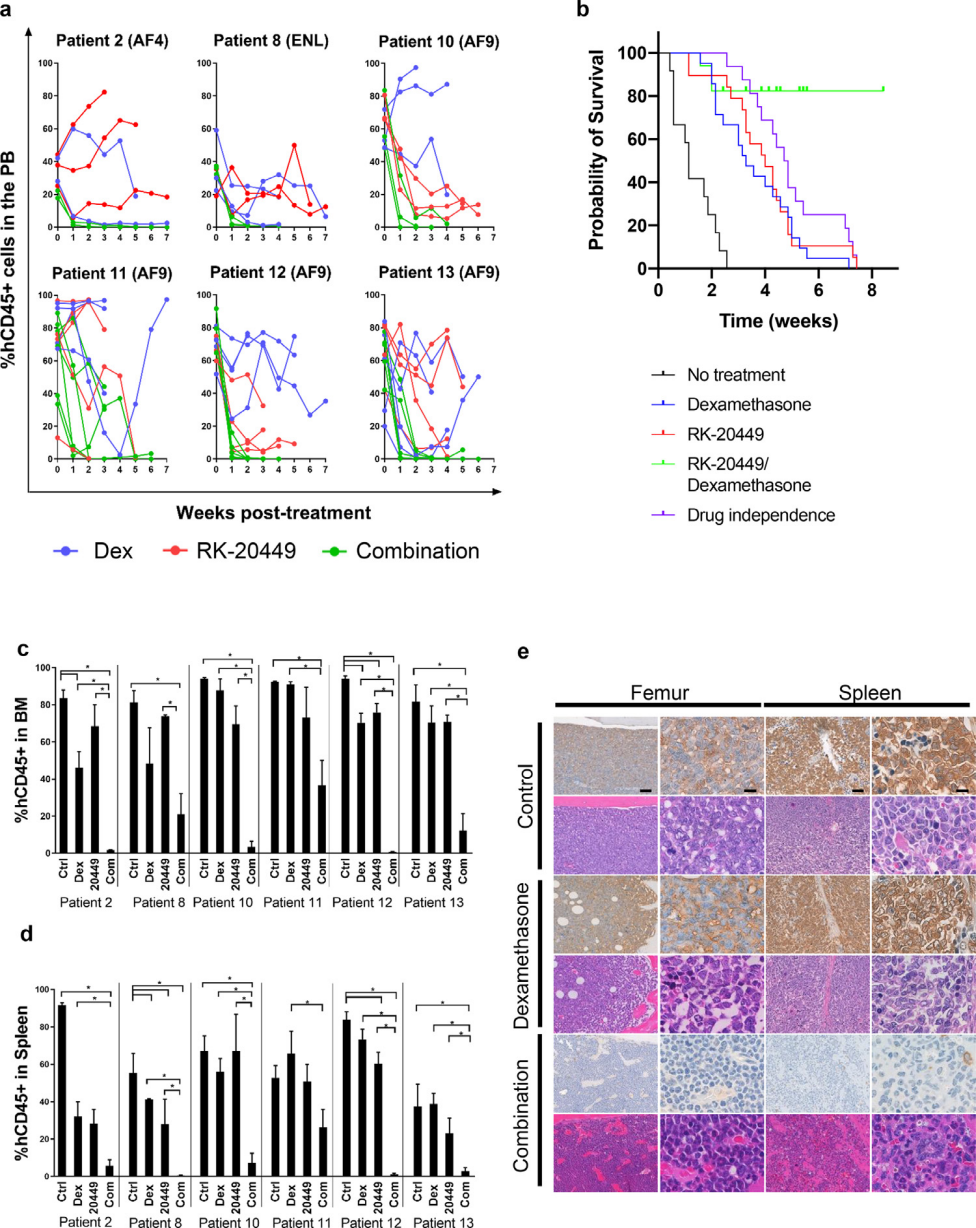
**Elimination of human MLL-ALL cells by combination treatment *in vivo*.** Treatment effect of dexamethasone alone, RK-20449 alone and combination of the two on LICS from MLL-ALL-engrafted recipient mice. Recipients engrafted with MLL-ALL cells were developed from one AF4 patient, one ENL patient and four different MLL-AF9 patients (see Table S1). Control treated recipients *n*=21 (Pt.2 *n*=3, Pt.8 *n*=3, Pt.10 *n*=3, Pt.11 *n*=4, Pt.12 *n*=5, Pt.13 *n*=3) dexamethasone-treated recipients *n*=21 (Pt.2 *n*=2, Pt.8 *n*=3, Pt.10 *n*=3, Pt.11 *n*=4, Pt.12 *n*=4, Pt.13 *n*=5); RK-20449-treated recipients *n*=21 (Pt.2 *n*=3, Pt 8 *n*=2, Pt.10 *n*=3, Pt.11 *n*=5, Pt.12 *n*=4, Pt.13 *n*=4); Combination-treated recipients *n*=24 (Pt.2 *n*=2, Pt.8 *n*=3, Pt.10 *n*=3, Pt.11 *n*=6, Pt.12 *n*=5, Pt.13 *n*=5). Both drugs were administrated in a concentration of 30 mg/kg of which dexamethasone was administrated once a day and RK-20449 twice a day. (a) *In vivo* effect of dexamethasone alone (blue line), RK-20449 alone (red line) and combination of the two drugs (green line) on hCD45+ cells in the PB of MLL-ALL-engrafted recipient mice over time. (b) Kaplan–Meier survival probability curves showing overall survival probability in untreated mice (black line), MLL-ALL recipient mice treated with dexamethasone (blue line), RK-20449 (red line), or a combination of the two drugs (green line). Control recipients *n*=13 (Pt.2 *n*=2, Pt.8 *n*=1, Pt.10 *n*=1, Pt.11 *n*=3, Pt.12 *n*=4, Pt.13 *n*=1), dexamethasone-treated recipients *n*=21 (Pt.2 *n*=1, Pt.8 *n*=3, Pt.10 *n*=3, Pt.11 *n*=4, Pt.12 *n*=3, Pt.13 *n*=7), RK-20449-treated recipients *n*=19 (Pt.2 *n*=2, Pt.8 *n*=1, Pt.10 *n*=3, Pt.11 *n*=4, Pt.12 *n*=5, Pt.13 *n*=4), combination-treated recipients n=16 (Pt.2 *n*=3, Pt.8 *n*=1, Pt.10 *n*=2, Pt.11 *n*=4, Pt.12 *n*=3, Pt.13 *n*=4). An event was defined as when a recipient became moribund and % human MLL-ALL cells in the BM was greater than 30% at the time of sacrifice. Recipients were censored if % human MLL-ALL cells in both BM and peripheral blood were less than 10% after treatment course. Combination of RK-20449 and dexamethasone resulted in significantly prolonged survival compared with estimated additive effect of RK-20449 and dexamethasone (shown as drug independence, purple line) (*p*=0.0245 by log-rank test), indicating synergistic effect of RK-20449 and dexamethasone on survival. (c–e) The effect of dexamethasone alone, RK-20449 alone and combination on hCD45+ cells in (c and e) BM and (d and e) spleen of MLL-ALL recipients treated for 4 to 11 weeks. (c and d) Remaining percentages of hCD45+ MLL-ALL cells in (c) BM and (d) spleen after treatment period. Results are expressed as ± 1 standard error of the mean with *P<0.05 by two-tailed *T*-test. (e) Representative photomicrographs show location of the remaining hCD45+ cells in BM and spleen of NSG recipients engrafted with MLL-ALL cells from patient 12 after the treatment. Tissue sections were stained with hematoxylin and immune-stained for hCD45 or with hematoxylin and eosin. Scale bar in the left panels means 50 mm and that in the right panels depicts 10 mm.

Since we could not eliminate MLL-ALL cells with dexamethasone alone, we assessed whether additional inhibition of SFKs and FLT3 leads to more efficient killing of leukemic cells in vivo. We performed *in vivo* therapeutic experiments using RK-20449, an inhibitor of SFKs and FLT3, alone or in combination with dexamethasone. For the treatment experiments, we created MLL-ALL-engrafted NSG recipient mice using leukaemia cells derived from six MLL-ALL patients. After human MLL-ALL chimerism reached 20% or greater, recipients were treated with RK-20449 (30 mg/kg twice a day), alone or in combination with dexamethasone (30 mg/kg once daily). We analyzed the frequency of hCD45+ leukemic cells with flow cytometry, and found that dexamethasone and RK-20449 as single compounds were not able to eradicate human MLL-rearranged leukaemia cells from PB whereas combination of the two compounds led to significant reduction of hCD45+ cells in the PB of MLL-ALL-engrafted mice (Fig. 3a and Table S2) and prolonged survival of treated mice (Fig. 3b). Using Bliss-based algorithm assessing in vivo synergist effect [36], we found combination treatment using Dexamethasone and RK-20449 displayed synergistic effect in achieving prolonged survival of MLL-ALL engrafted recipients. After 2 to 7 weeks of treatment, we analyzed BM and spleen of the recipients to assess the therapeutic effect. In MLL-ALL engrafted mice treated with either RK-20449 alone or dexamethasone alone, we found substantial number of residual hCD45+ cells in BM and spleen. In contrast, combined treatment with RK-20449 and dexamethasone resulted in a significant reduction with nearly undetectable levels of hCD45+ cells in the BM and spleen of the recipients (Fig. 3c, d, and Table S2). Immunohistochemical staining for hCD45 in the recipient BM, spleen, kidney and liver confirmed the in vivo elimination of patient leukaemic cells. In the untreated mice, the majority of BM cells were hCD45+ leukaemia cells, with infiltration of hCD45+ cells in spleen, liver, and kidney. Mice treated with RK-20449 alone or dexamethasone alone showed residual hCD45+ leukemic cells in each of organs examined. In contrast, human cells were nearly completely eliminated from these organs in recipients treated with RK-20449 and dexamethasone (Fig. 3e, S2, and S3). Furthermore, the BM of all MLL-ALL recipients showed recovery of murine hematopoiesis concurrent with the elimination of human leukaemia cells during treatment with dexamethasone and RK-20449, suggesting that combination treatment spares normal haematopoiesis to some extent (Fig. 3e, S2, and S3). These findings demonstrate the efficacy and safety of RK-20449 combined with dexamethasone against human MLL-ALL *in vivo*. However, NSG mice engrafted with MLL-ALL derived from Patient 11 showed the most remaining leukaemia cells after combination treatment, in PB (Fig. 3a), BM and spleen (Fig. 3c and d) compared to the recipients engrafted with the other five cases indicating that leukemic cells of Patient 11 are resitant even to the combination treatment. Immunohistochemical staining of various organs confirm these findings (Fig. 3e, S2 and S3). Therefore, we went on to identify additional mechanisms that render Patient 11-derived MLL-ALL cells resistant to combined treatment with RK-20449 and dexamethasone.

### 3.4 BCL-2 inhibition induces apoptosis in MLL-ALL cells resistant to combination therapy, *in vitro* and *in vivo*

**Fig. 4.**
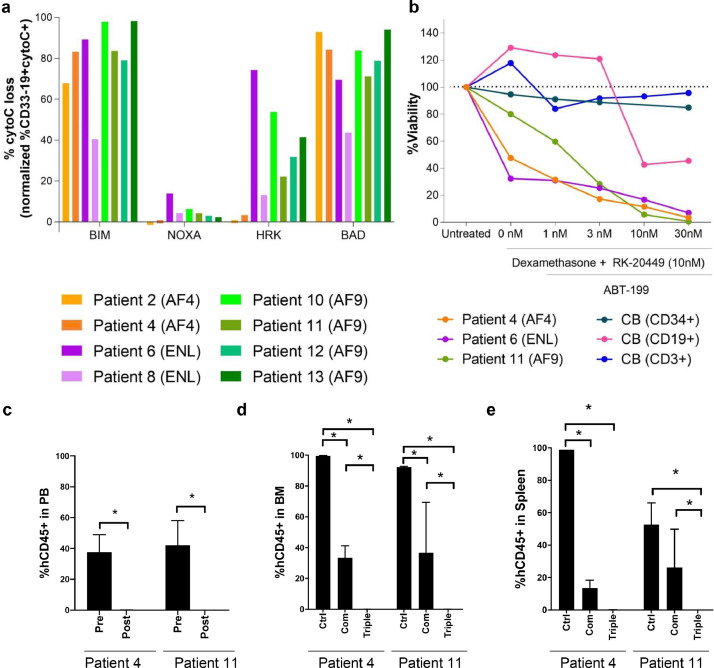
**Inhibition of BCL-2 induces apoptosis in resistant MLL-ALL cells.** (a) BH3 profiling in CD33-19+ BM cells of MLL-ALL recipients. Recipients were engrafted with MLL-ALL cells obtained from 8 different MLL-ALL patients (see Table S1). AF4 recipients *n*=2 (Pt.2 and 4), ENL recipients *n*= 2 (Pt.6 and 8), AF9 recipients *n*=4 (Pt.10, 11, 12 and, 13). The effect of BH3 peptides, BIM, NOXA, HRK and BAD were tested on the MLL-ALL cells. (b) *In vitro* effect of ABT-199 treatment in combination with dexamethasone and RK-20449 on hCD45+ MLL-ALL spleen and normal haematopoietic cord blood (Stem cells (CD34+), B-cells (CD19+) and T-cells (CD3+)) cells. Spleen cells were obtained from MLL-ALL recipients engrafted with MLL-ALL cells from 3 different patients (Pt.4, 6, and 11). Normal haematopoietic cells were obtained from human cord blood samples. Dexamethasone and RK-20449 were administrated in a concentration of 10 nM. ABT-199 was administrated in 4 different concentrations (1 nM, 3 nM, 10 nM and 30 nM) and incubated for 3 days at 37 C. (C–F) *In vivo* effect of combination-treatment with dexamethasone and RK-20449 and triple-treatment with dexamethasone, RK-20449 and ABT-199 on hCD45+ cells of MLL-ALL engrafted NSG mice. All drugs were administrated in a concentration of 30 mg/kg of which dexamethasone i.p. once a day, RK-20449 i.p. twice a day and ABT-199 orally once a day. Recipients engrafted with MLL-ALL cells were developed from Patient 4 (AF4) and 11 (AF9) (see Table S1). Control treated recipients *n*=6 (Pt.4 *n*=2, Pt.11 *n*=4), Combination treated recipients *n*=9 (Patient 4 *n*=3, Patient 11 *n*=6), Triple treated recipients *n*=6 (Pt.4 *n*=3, Pt.11 *n*=3). (c) Pre- and post-treatment levels of hCD45+ cells in the PB of MLL-ALL-engrafted recipient mice after triple-treatment. The effect of combination and triple-treatment on hCD45+ cells in (d) BM and (e) spleen of MLL-ALL recipients. Results are expressed as ± 1 standard error of the mean with *P<0.05 by two-tailed *T*-test.

**Fig. 5.**
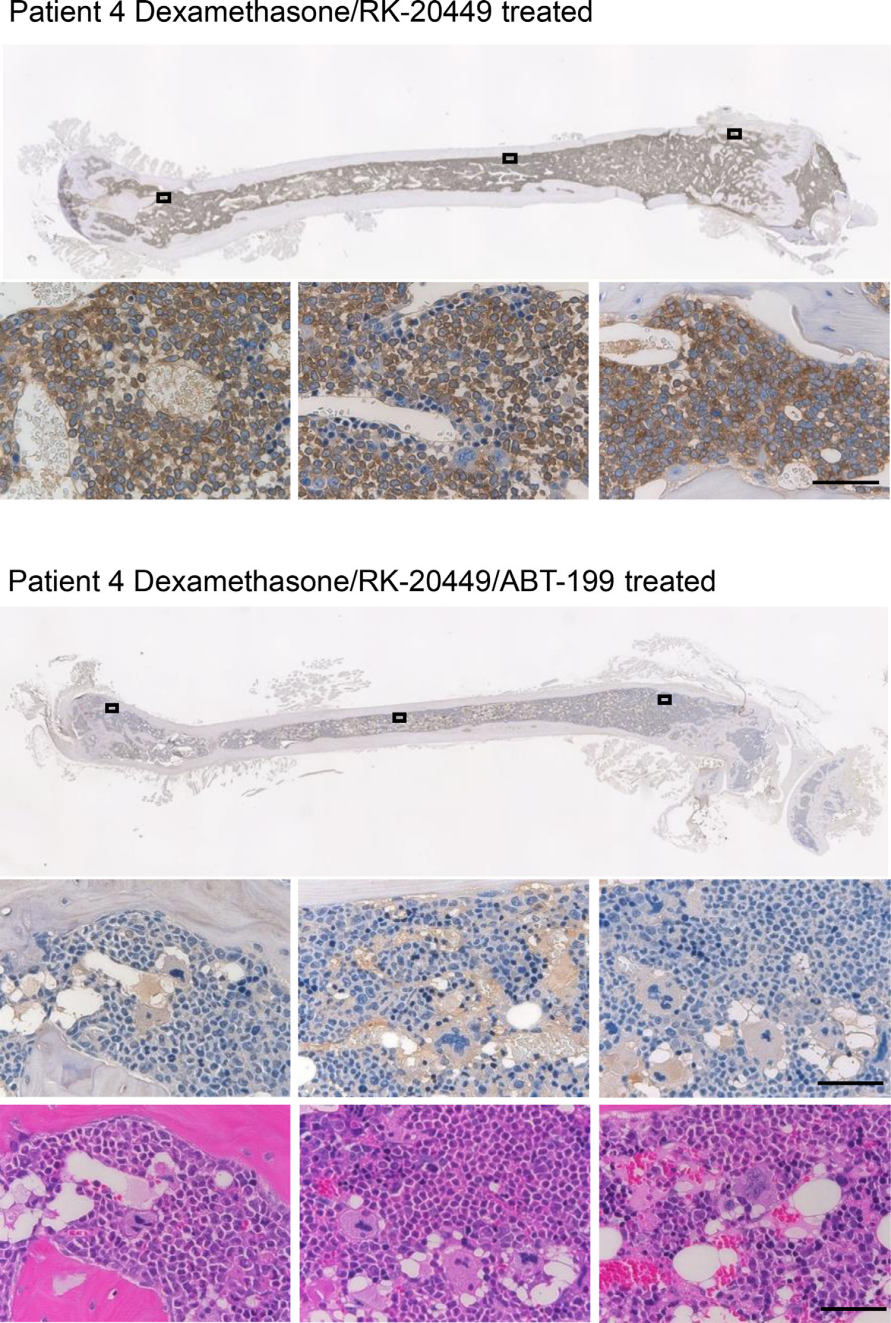
**Elimination of MLL-ALL cells from the BM of a Patient 4 engrafted recipient that showed resistant to combination treatment.** Representative photomicrographs showing the location of remaining hCD45+ MLL-ALL cells in BM of Patient 4 engrafted NSG recipients after the treatment. The recipients were treated with a combination of dexamethasone and RK-20449 (upper) or with a triple combination of dexamethasone, RK-20449 and ABT-199 (lower). All drugs were administrated in a concentration of 30 mg/kg dexamethasone i.p. once a day, 30 mg/kg RK-20449 i.p. twice a day and ABT-199 orally once a day. Femurs were immune-stained for hCD45 or stained with hematoxylin and eosin. Bars represent 50 μm.

As an additional target in MLL-ALL cells resistant to the combination treatment, we focused on anti-apoptosis pathway which was enriched in MLL-ALL-initiating cells compared with normal HSPCs (Fig. 2a). Altered expression of apoptotic genes can disturb the balance between pro- and anti-apoptotic proteins which thereby affects cell fate decision of MLL-ALL cells. The Letai lab developed Bcl-2 homology domain 3 (BH3) profiling [35] as a functional tool to get mechanistic insight into the mitochondrial apoptotic pathway, by measuring mitochondrial permeabilization after exposing cells to BH3 peptides. To identify key BH3 peptides that are responsible for the treatment resistance, we examined levels of mitochondrial priming in human MLL-ALL cells to apoptosis by BH3 profiling. In the BH3 profiling, we exposed eight cases of human MLL-ALL cells to BH3 sensitizer peptides NOXA, HRK and BAD as well as pro-apoptotic activator peptide, BIM. We found that in all cases, exposure to BIM resulted in mitochondrial cytochrome C release in human MLL-ALL cells. Furthermore, among three BH3 selective peptides, exposure to BAD resulted in the most efficient mitochondrial cytochrome C release, suggesting that human MLL-ALL cells depend on BCL-2 for survival (Fig. 4a). We therefore proceeded to assess whether inhibition of the BCL-2 anti-apoptotic pathway overcomes resistance to RK-20449/dexamethasone combination treatment, using MLL-ALL cells that survived dexamethasone/RK-20449 combination treatment (Patients 4, 6 and 11) (See Table S2). MLL-ALL cells obtained from engrafted recipients were incubated with dexamethasone and RK-20449 plus various concentrations of ABT-199, a BCL-2 inhibitor (Fig. 4b) [37,38]. We found that the addition of ABT-199 eliminated MLL-ALL cells resistant to dexamethasone and RK-20449*.* At the same time, human CB-derived HSPCs (CD34+) and mature T cells (CD3+) were not affected by treatment with dexamethasone, RK-20449 and ABT-199 while mature B-cells (CD19+) cells showed expected reduction by the addition of ABT-199 (Fig. 4b) [39]. ABT-199 alone treatment resulted in temporal reduction of MLL-ALL cells in PB, but residual leukemic cells in the BM and spleen and didn't prolong survival of ALL-engrafted recipients (Fig. S4a–d). However, oral administration of ABT-199 in addition to the injection of 30 mg/kg of dexamethasone and RK-20449 to the MLL-ALL recipients (Patients 4 and 11) resulted in complete elimination of hCD45+ cells in the PB (Fig. 4c), BM (Fig. 4d) and spleen (Fig. 4e). These findings were confirmed by immunohistochemistry of BM from patient 4 engrafted NSG recipients (Fig. 5).

## 4 Discussion

Currently, GC such as dexamethasone has been used as one of the key drugs for treating patients with lymphocytic leukaemia or malignant lymphoma, as they induce apoptosis in malignant lymphoid cells [40]. Although GCs are more effective in lymphoid than myeloid diseases [41], relapse of leukaemia in infants with MLL-ALL after treatment with GCs remains as a significant problem. Earlier studies investigated resistance of MLL-ALL cells to GCs *in vitro* [[18], [19], [20], [21],^42^,^43^]. More recently, Kerstjens et al. developed a MLL-ALL cell line xenograft model, using a RAS-mutated MLL-rearranged infant ALL cell line, KOPN8, to investigate GC-resistance in MLL-ALL cells [44]. In addition to the published study, we further aimed to address inter-patient heterogeneity and patient-specific treatment resistance. In this study, we successfully developed an *in vivo* therapeutic model by xenogeneic transplantation of primary patient-derived cells into NSG recipients, which mimics the resistance of infant MLL-ALL cells to dexamethasone. Even from patients with a low peripheral disease burden and little to no signs of extra-medullary involvement (Patients 11 and 13), we were able to recapitulate GC-resistance in MLL-ALL engrafted mice.

To understand the mechanism of GC-resistance in MLL-ALL patients, we first searched for enriched pathways in primary patient MLL-ALL cells compared with normal HSCs. Previous studies showed that inhibition of Src kinase [20] may overcome GC resistance in MLL-ALL cells *in vitro*. In this study, we found that MLL-ALL cells showed elevated expression of HCK and BLK, members of SFKs, compared with normal HSCs. In addition, it was previously demonstrated that knockdown of S100A10 blocks Annexin A2 phosphorylation and subsequently leads to GC-sensitization in MLL-rearranged ALL [21]. We found expression of both S100A10 and ANXA2 to be elevated in LICs of MLL-ALL patients compared to normal HSPCs. Furthermore, a recent study confirmed that MLL-leukaemia cells become rapidly resistant to dexamethasone *in vitro* while becoming more sensitive to kinase signaling. Especially, FLT3 signaling activity was increased in MLL-leukaemia cells leading to constitutive activation of FLT3 downstream signaling pathways [23]. Consistent with these findings, we observed enrichment of several FLT3 and SFK downstream pathways which we hypothesize play an important role in GC-resistance. First, the MAPK signaling pathway is known to be activated by FLT3-ligand (FL) stimulation in MLL-rearranged leukaemia cells [45]. Moreover, this pathway is believed to be activated via HCK phosphorylation leading to cell proliferation [46]. Previous studies reported that MAPK signaling plays an important role in development of GC-resistance in pediatric ALL cells and that inhibition of this pathway restores GC-sensitivity [42,44,47]. Furthermore, Delgado-Martin et al. suggest that IL7R/JAK/STAT inhibition sensitizes otherwise GC-resistant T-ALL cells to GCs [48]. The JAK-STAT pathway is believed to be one of the FLT3 downstream pathways in leukaemia [8,49] and the claim was strengthen by a report of upregulated STAT5 phosphorylation following FL stimulation in MLL-rearranged leukaemia [45]. In addition, STAT5 binds phosphorylated SFK which then leads to cytoplasmic signaling in myeloid leukaemia's [50]. SFK inhibitors block this constitutive activation of STAT5 in (AML) cells [51]. Finally, inhibition of another FLT3 downstream pathway [8,45,49], the PI3K-Akt signaling pathway, reverses resistance of T-ALL [52] and MLL-ALL [19] cells to GCs. Inhibition of HCK reduces the PI3K-Akt, but also the MAPK signaling pathway, in cells with upregulated HCK expression and thereby HCK becomes a potential drug target in leukaemia [53]. Taken together, SFK and FLT3 signaling seems to drive MLL-ALL via several pathways which are cooperatively involved in GC-resistance. Therefore, in this study we aimed to target both kinases on MLL-ALL cells with RK-20449. The findings would possibly be applied to non-MLL paediatric leukaemia, since genes involved in downstream of SFK or FLT3 signalling such as HCK, IL7R, and S100A10 were also upregulated in leukaemia cells with other genetic abnormalities including the Philadelphia chromosome, ETV6-RUNX1 translocation, and t(5;15) translocation.

MLL-ALL engrafted recipients were treated with dexamethasone and RK-20449. Although each drug showed some single agent activity, the effect tended to be incomplete or of limited duration. Combination therapy, however, rapidly reduced the number of MLL-ALL cells from the circulation and also eliminated the leukaemia cells from BM and spleen in the majority of engrafted mice. In addition, MLL-ALL cells infiltrating the liver and kidney were successfully cleared by the combination treatment. These findings suggest that inhibition of SFKs and FLT3, with subsequent reduction of corresponding downstream pathways, reverses GC-resistance in infant MLL-ALL cells.

However, in 3 out of 8 cases, we found residual leukaemia cells in the recipient BM and spleen after the combination treatment with dexamethasone and RK-20449. In the aim of clarifying which BH3 peptide MLL-ALL cells depend for survival, we performed BH3 profiling showing that MLL-ALL cells were dependent upon Bcl-XL and Bcl-2 for their survival. However, Bcl-XL inhibition is known to induce thrombocytopenia in patients with lymphoid malignancies [54]. Consistent with our findings, an earlier study showed that MLL-ALL cell lines express high levels of Bcl-2 proteins and these cell lines as well as patient-derived MLL-ALL xenografts were highly sensitive to the Bcl-2 inhibitor ABT-199 [55]. We found that the triple-treatment strategy using dexamethasone, RK-20449 and ABT-199 was highly effective in targeting the otherwise resistant MLL-ALL cells *in vitro* and *vivo*. Moreover, normal human HSCs and T-cells were not affected by this drug treatment protocol. On the other hand, triple-treatment was cytotoxic for normal mature B cells *in vitro*; this is consistent with a report showing that both malignant and normal mature B cells, but not normal precursor B-cells or myeloid cells, are affected by ABT-199 treatment [39]. Our findings are consistent with recent studies showing the effectiveness of inhibiting overexpressed kinases and Bcl-2 proteins in leukaemia [[56], [57], [58]]. In addition, Goossens et al., showed that JAK-STAT overexpression leads to increased Bcl-2 transcription and thereby inhibits the GC-induced intrinsic apoptosis pathway in leukaemia cells [59]. Therefore, we believe that combination treatment of the multiple kinase inhibitor RK-20449 and the Bcl-2 inhibitor ABT-199 overcomes GC-resistance in MLL-ALL, offering a new effective therapeutic strategy for this high-risk disease.

## Data sharing statement

The above microarray data can be accessed without any restrictions in RefDIC database (http://refdic.rcai.riken.jp/welcome.cgi) under the following accession numbers: RSM08287, RSM08288, RSM08289, RSM08292, RSM08293, RSM08294, RSM08295, RSM08395, RSM09150, RSM09151, RSM09152, RSM09189, RSM09190, RSM09536, RSM09537, RSM13489, RSM13490, RSM13491, RSM29891, RSM29892, RSM29893, RSM29894, RSM29895, RSM29896, RSM29897, RSM29898.

## Declaration of Interests

The authors declare that they have no competing interests.

## Appendix Supplementary materials

**Supplemental figure 1 – Upregulated kinase activity in acute lymphoblastic leukaemias.** (a) Heatmap showing GCRMA-obtained transcript expression levels in ALLs with and without MLL translocation. MLL-ALL patients *n*=3 (Pt.12, 13 (AF9) and 20 (AF4)) and non-MLL ALL patients *n*=5 (Pt.19 (unknown karyotype), 15 (Ph+ALL), 16, 17 (ETV6-RUNX1) and 18 (t5;15)). Key transcripts such as HCK, ANXA2 and IL7R are found to be highly expressed in non-MLL patients, which is comparable with MLL-ALL patients. (b) Similar to MLL-ALL, high phosphorylation levels of NF-κB, S6, 4EBP1 & AKT were found in hCD45+ cells from non-MLL acute lymphoblastic leukaemia engrafted NSG-recipients of one Philadelphia-positive ALL patient (Pt.15), one ETV6-RUNX1 ALL patient (Patient 16), one t(5;15) ALL patient (Pt.18), and one ALL patient with unknown karyotype (Patient 19) compared to two freshly isolated normal CD34+ cord blood samples. (c) GCRMA-obtained expression values of the key signaling proteins, NF-κB (*RELA*), S6 (*RPS6*), 4EBP1 (*EIF4EBP1*), and AKT (*AKT1, AKT2, AKT3*) at the RNA level in three normal CD34+ cord blood samples and 11 LIC MLL-ALL samples (Pt.1-5 (AF4), 6-7, 9 (ENL) and 10-11, and 14 (AF9)). Transcript levels of *RELA, RPS6, AKT1* and *AKT2* are not different between CD34+ cord blood samples and LIC MLL-ALL cells. Transcript levels of *EIF4EBP1* and *AKT3* are downregulated in MLL-ALL. This indicates that upregulation of the kinase signaling pathways are mostly regulated by post-translational modifications. Results are expressed as ± 1 standard error of the mean with *P<0.05 by two-tailed T-test. **Supplemental figure 2 – Treatment with RK-20449 and dexamethasone eliminates MLL leukemic cells in the BM, spleen, kidney and liver of NSG recipients.** The effect of dexamethasone, RK-20449 and combination on infiltrated hCD45+ cells in BM, spleen, kidney and liver. Tissue sections were stained with hematoxylin and immune-stained for hCD45. Bars represent 100 μm. **Supplemental figure 3 – The effect of combination treatment on the recovery of the murine hematopoiesis.** Femurs of untreated and combination-treated MLL-ALL recipient mice were stained with hematoxylin and eosin. Bars represent 100 μm and 20 μm. **Supplemental figure 4 – Single treatment with ABT-199 has a limited effect on MLL-ALL engrafted NSG recipients.** Recipients engrafted with MLL-ALL cells were developed from one AF4 patient, one ENL patient and two different MLL-AF9 patients to assess ABT-199 treatment effect against MLL-ALL (see table S1, no treatment control *n*=13 (Pt.2 *n*=3, Pt.6 *n*= 2, Pt.12 *n*=5, Pt.13 *n*=3), ABT-199 treatment *n*=7 (Pt.2 *n*=2, Pt.6 *n*=1, Pt.12 *n*=3, Pt.13 *n*=1). ABT-199 was orally administrated in a concentration of 30 kg/mg (Pt.2 and 12) or 70kg/mg (Pt.6 and 13) once a day. (a) *In vivo* effect of ABT-199 on hCD45+ cells in the PB of MLL-ALL-engrafted recipient mice over time. (b and c) The effect of ABT-199 on hCD45+ cells in (b) BM and (c) spleen of MLL-ALL recipients treated for 4 weeks. (d) The overall survival probability in MLL-ALL recipients treated with ABT-199 *n*=7 (Pt.2 *n*=2, Pt.6 *n*=1, Pt.12 *n*=3, Pt.13 *n*=1). Same Kaplan Meier survival probability curves for dexamethasone and RK-20449 as in Fig. 3b are shown. Ticks mark the censoring times of successfully treated recipient mice who showed % human MLL-ALL cells in both BM and PB less than 10%. Image, application 1

Image, application 2

Image, application 3
