# Supplementary figures and images for: Targeting critical kinases and anti-apoptotic molecules overcomes steroid resistance in MLL-rearranged leukaemia
Source: eBioMedicine. 2021 Feb 10;64:103235. doi: 10.1016/j.ebiom.2021.103235 (PMC7878180; doi:10.1016/j.ebiom.2021.103235)

Figure S1

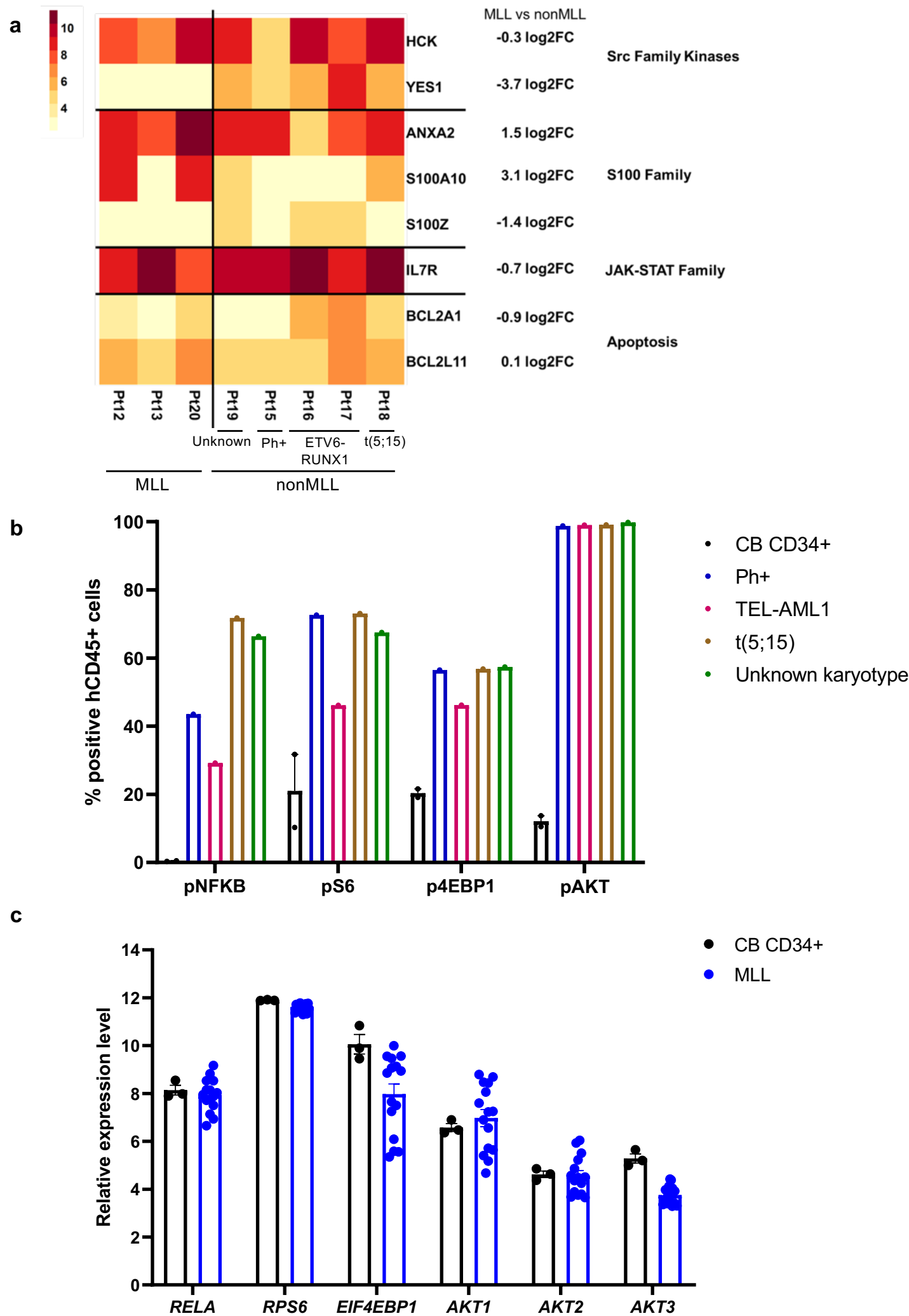

Figure S2

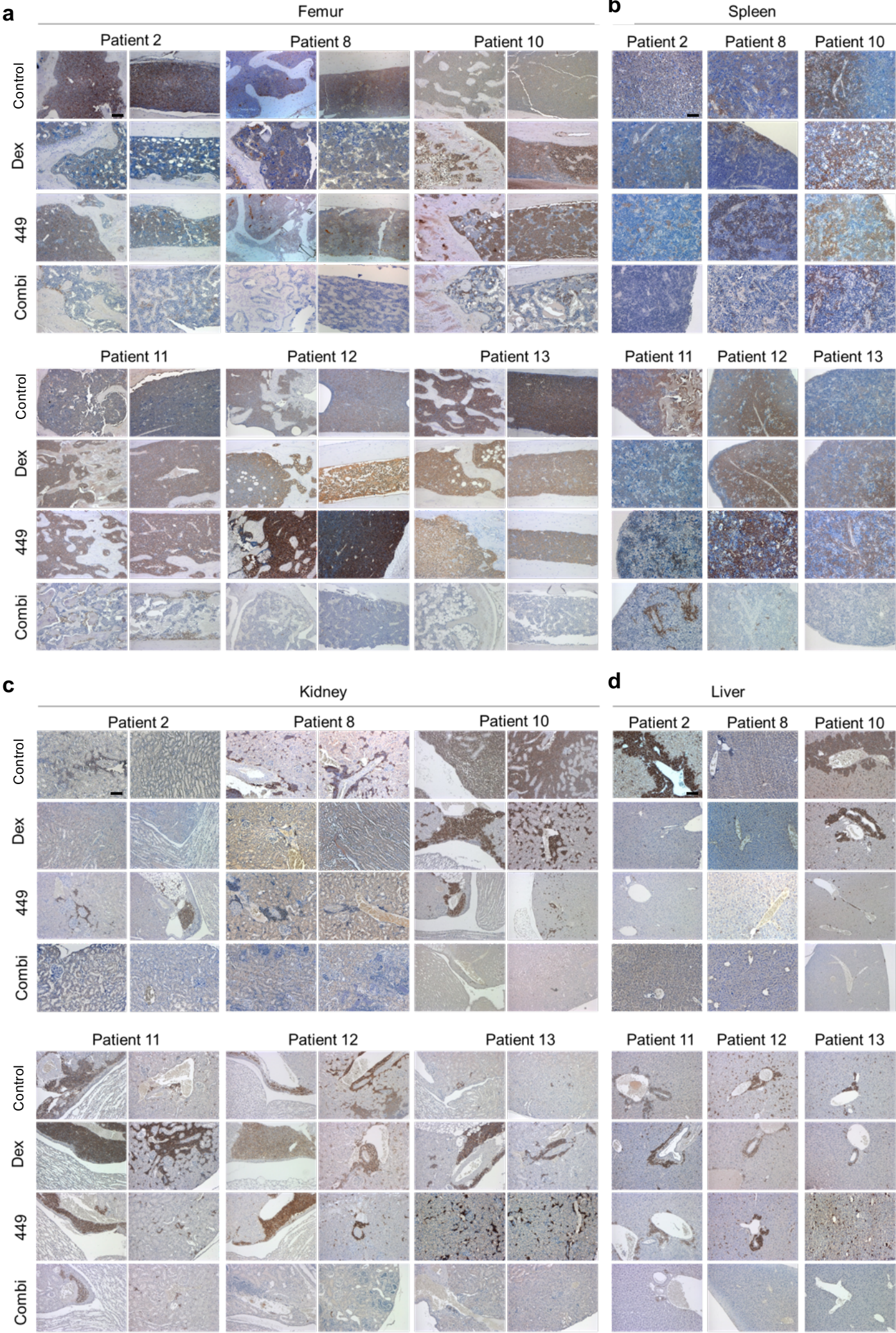

Figure S3

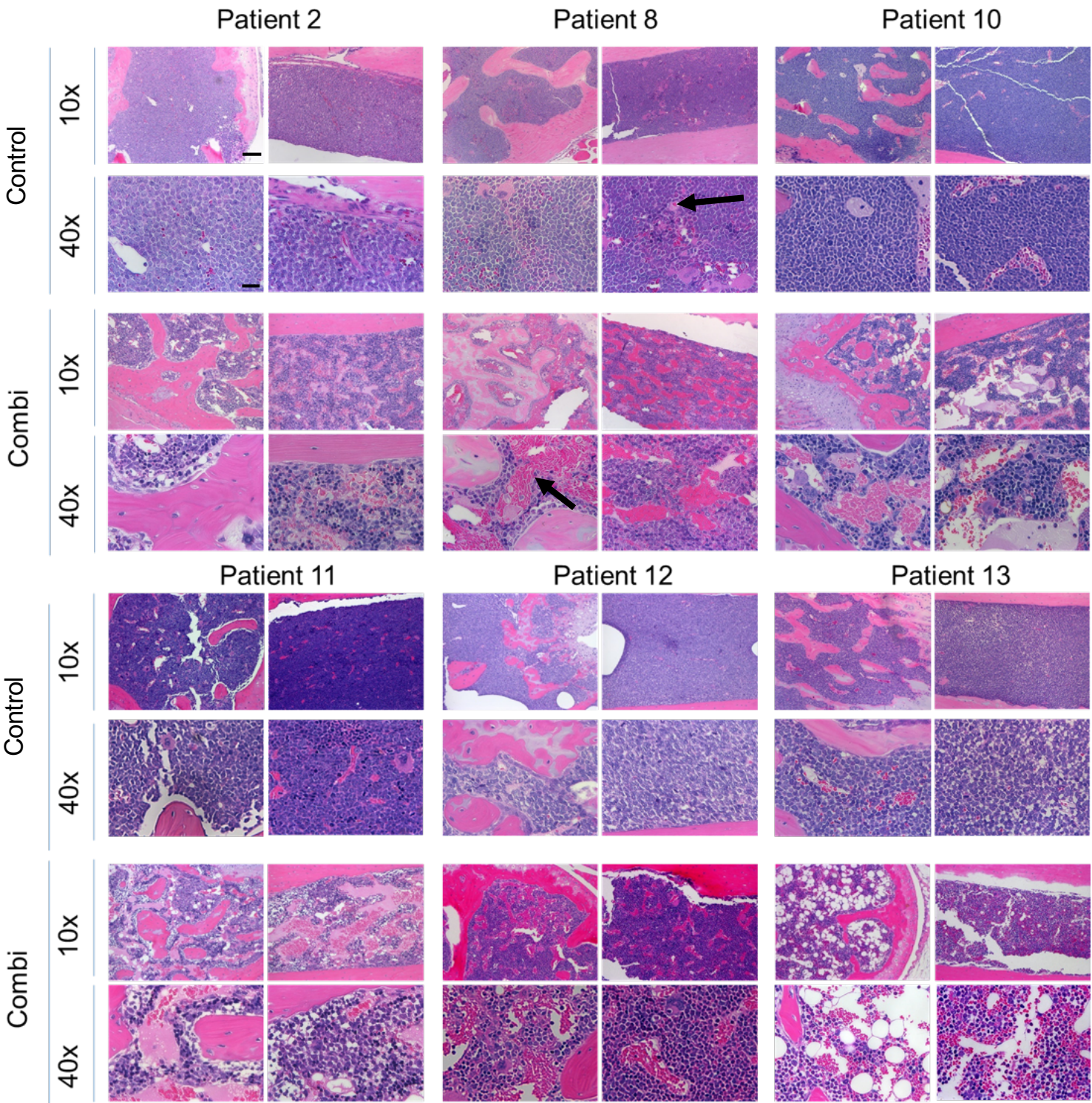

Figure S4

a

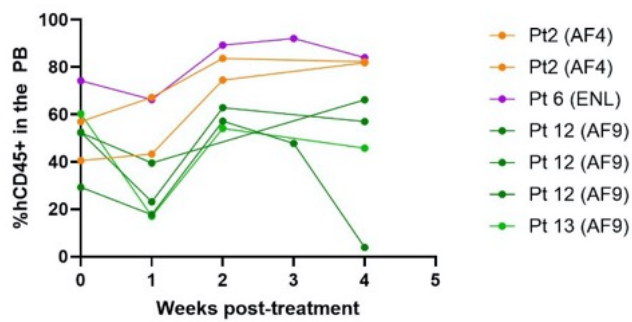

b

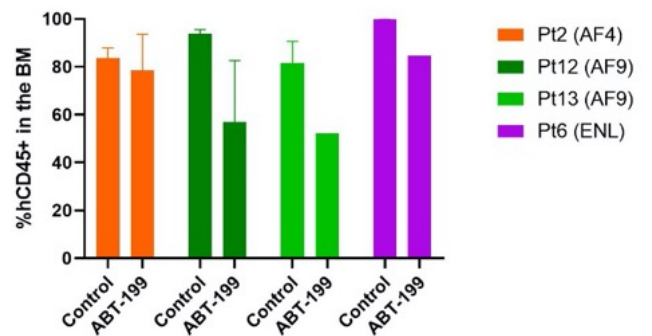

c

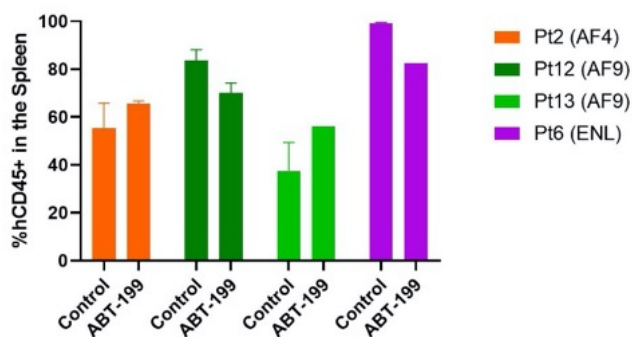

d

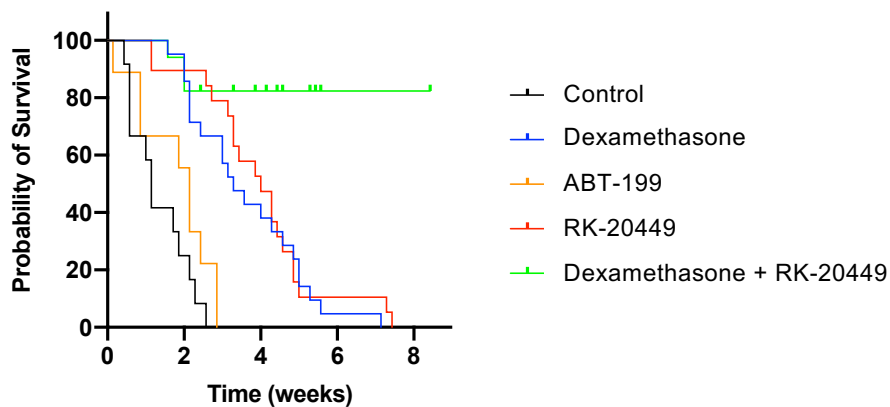

Supplement: Supplementary file 1 — Supplemental figure 1 – Upregulated kinase activity in acute lymphoblastic leukaemias. (a) Heatmap showing GCRMA-obtained transcript expression levels in ALLs with and without MLL translocation. MLL-ALL patients n=3 (Pt.12, 13 (AF9) and 20 (AF4)) and non-MLL ALL patients n=5 (Pt.19 (unknown karyotype), 15 (Ph+ALL), 16, 17 (ETV6-RUNX1) and 18 (t5;15)). Key transcripts such as HCK, ANXA2 and IL7R are found to be highly expressed in non-MLL patients, which is comparable with MLL-ALL patients. (b) Similar to MLL-ALL, high phosphorylation levels of NF-κB, S6, 4EBP1 & AKT were found in hCD45+ cells from non-MLL acute lymphoblastic leukaemia engrafted NSG-recipients of one Philadelphia-positive ALL patient (Pt.15), one ETV6-RUNX1 ALL patient (Patient 16), one t(5;15) ALL patient (Pt.18), and one ALL patient with unknown karyotype (Patient 19) compared to two freshly isolated normal CD34+ cord blood samples. (c) GCRMA-obtained expression values of the key signaling proteins, NF-κB (RELA), S6 (RPS6), 4EBP1 (EIF4EBP1), and AKT (AKT1, AKT2, AKT3) at the RNA level in three normal CD34+ cord blood samples and 11 LIC MLL-ALL samples (Pt.1-5 (AF4), 6-7, 9 (ENL) and 10-11, and 14 (AF9)). Transcript levels of RELA, RPS6, AKT1 and AKT2 are not different between CD34+ cord blood samples and LIC MLL-ALL cells. Transcript levels of EIF4EBP1 and AKT3 are downregulated in MLL-ALL. This indicates that upregulation of the kinase signaling pathways are mostly regulated by post-translational modifications. Results are expressed as ± 1 standard error of the mean with *P<0.05 by two-tailed T-test. Supplemental figure 2 – Treatment with RK-20449 and dexamethasone eliminates MLL leukemic cells in the BM, spleen, kidney and liver of NSG recipients. The effect of dexamethasone, RK-20449 and combination on infiltrated hCD45+ cells in BM, spleen, kidney and liver. Tissue sections were stained with hematoxylin and immune-stained for hCD45. Bars represent 100 μm. Supplemental figure 3 – The effect of c [file mmc1.pdf]
